# Osmotic dehydration and convective drying of kiwifruit (*Actinidia deliciosa*) – The influence of ultrasound on process kinetics and product quality

**DOI:** 10.1016/j.ultsonch.2020.105377

**Published:** 2020-10-28

**Authors:** Joanna Kroehnke, Justyna Szadzińska, Elżbieta Radziejewska-Kubzdela, Roża Biegańska-Marecik, Grzegorz Musielak, Dominik Mierzwa

**Affiliations:** a Poznań University of Technology, Institute of Technology and Chemical Engineering, Department of Process Engineering, ul. Berdychowo 4, 60-965 Poznań, Poland; b Poznan University of Life Sciences, Institute of Food Technology of Plant Origin, ul. Wojska Polskiego 31, 60-624 Poznań, Poland

**Keywords:** Intensification of mass transfer, Ultrasound heating effect, Color, Water activity, Carotenoids, Polyphenols

## Abstract

The aim of this study was to assess the impact of ultrasound on two subsequent processes – initial osmotic dehydration and convective drying of kiwifruit (Actinidia deliciosa). The effect of ultrasound (at a frequency of 25 kHz) was assessed both in terms of process kinetics and product quality. During the study, three different osmotic agents were used – erythritol, sorbitol, and sucrose – in an aqueous solution at a concentration of 50% (w/w). The essential kinetic parameters were analyzed – water loss and increase of dry matter during osmotic dehydration, and evolution of moisture content and temperature of samples during convective drying (drying curves). Product quality was assessed on the basis of color, water activity, and content of relevant bioactive components – polyphenols and carotenoids. It was found that the application of ultrasound during osmotic dehydration resulted in higher water loss and solid gain. This surely results from the phenomena occurring during the propagation of the elastic waves in the liquid medium (mainly related to cavitation) and enhancement of the mass transfer. The use of ultrasound during convective drying also had a positive effect on the kinetics of this process. In most cases, during the ultrasonically assisted drying operations, a significant increase in drying rate was observed, leading to a reduction in drying time. This may be due to the intensification of both heat and mass transfer as a result of the mechanical and thermal effects of ultrasound. The assessment of product quality showed that the use of sugar alcohols was a good alternative to sucrose, and ultrasound-assisted convective drying increased the retention of valuable carotenoids and polyphenols. Moreover, in all dried kiwifruit slices, water activity was below 0.6, which can guarantee the microbiological stability of the tested samples.

## Highlights

• Use of ultrasound improves mass transport during osmotic dehydration of kiwifruit.
• Erythritol is the most effective osmoactive agent in 30 minutes of dehydration.
• Ultrasound-assisted convective drying (CDUS) of kiwifruit reduces drying time.
• Use of polyols and CDUS increases carotenoids and polyphenols retention.

## 1 Introduction

Convective drying (CD) is one of the most commonly used methods of preservation, especially for highly perishable products such as fruit and vegetables. The reduction of moisture content, thus water activity, during drying allows the microbial activity of the material to stabilize and significantly moderates other deteriorative processes such as enzymatic and non-enzymatic reactions, lipid oxidation, browning, etc. [1]. Unfortunately, CD can negatively affect the quality of the final product. Changes in color and taste, shrinkage and deformations, surface hardening, and loss of important nutrients are only examples of the negative effect of this processing method [2]. Moreover, due to the low energy efficiency of dryers, CD is usually time and energy consuming [3].

Osmotic dehydration (OD) is a common pretreatment process that reduces the negative effects of CD. According to the literature, the quality of the final products is visibly better if OD is used [4], [5]. The most popular osmotic agents are sugars (e.g. sucrose, fructose, glucose) for fruit, and salts (e.g. NaCl, KCl, CaCl) for vegetables and meat [4]. Since the WHO reports the negative effect of such substances on human health, and the reduction of their consumption is promoted [6], new osmotic agents are being sought. Another reason for this is that food producers have to adapt to the needs of the market, where the interest in food for diabetics or products with reduced sugar content is constantly increasing [7]. In this study, sorbitol and erythritol from the polyol group were selected as alternative compounds to sugars. These are low-digestible compounds with a low caloric value and glycemic index. However, as sorbitol is partly used by the microorganisms in the gastro-intestinal tract, its elevated intake may cause laxation [8]. Important features of erythritol are its relatively high stability in acidic and alkaline environments and against heat. Moreover, erythritol has been reported to have good digestibility without gastric discomfort [9].

The use of ultrasound (US) during osmotic dehydration (ultrasound-assisted OD, USOD) aims to accelerate the slow diffusional process of osmosis [10], [11]. It is known that mechanical waves such as US may significantly intensify heat and mass transfer due to several phenomena that occur during the passing of waves, e.g. stirring, microstreaming, pressure alteration, reduction of the boundary layer, etc. [12].

Kiwifruit, due to its nutritional qualities, is an important component in a healthy and balanced human diet. Because it is rich in vitamin C and phytonutrients including carotenoids, phenolics, and chlorophyll, kiwifruit has strong antioxidant properties. However, kiwifruit is highly perishable, and softens and loses nutritional value even in refrigerated conditions. Because of its short shelf-life, kiwifruit needs to be preserved, e.g. by freezing, canning, being processed into jams and juices, and drying [13], [14]. The most common method used to dry kiwifruit is hot air drying. Other options found in the literature are hot air microwave drying and microwave drying [15], vacuum drying [16], and infrared drying [17]. One technological solution for drying that has been studied recently is the combination of CD with additional energy sources, i.e. hybrid drying, such as using US [18]. Ultrasonic waves are a promising tool for strengthening hot air drying as they intensify the mass transfer through a high-frequency vibration generated inside the tissue of the material. This effect decreases internal moisture adhesion and reduces moisture diffusion resistance inside the material, which results in the remarkable intensification of water transfer [19].

In this study, an attempt to use sugar alcohols instead of sucrose was made. Kiwifruit (*Actinidia deliciosa*) was chosen as the experimental material. Different variants of osmotic processes were tested to analyze their kinetics and influence on the drying processes carried out by CD and CD combined with US. Additionally, the quality of the final products was assessed on the basis of overall color change, water activity, and the content of the bioactive compounds of carotenoids and polyphenols.

## 2 Material and methods

### 2.1 Sample preparation

Kiwifruit (*Actinidia deliciosa*) was bought at a local market and refrigerated at 277 K for at least 24 h. Before processing, the whole fruit was cut into slices, from which discs with a diameter of 32 mm and a height of 7 mm were cut out. A single sample had an initial mass of 6.4 ± 0.1 g.

### 2.2 Osmotic dehydration

In the first part, the kinetics of OD were analyzed to determine the effective time of dehydration. For this purpose, the kiwifruit samples were dehydrated in aqueous solutions of three different (analytically pure) osmotic agents: erythritol (ERY), sorbitol (SOR), and sucrose (SUC). The solutions were prepared by mixing the predetermined mass of the given compound in distilled water. The concentration of the solution was 50% (w/w). The hypertonic solution was poured into glass beakers (200 mL per beaker) that were next placed in a water bath at a temperature of 308 K to stabilize the temperature. After 15 min, the kiwifruit samples were immersed in the osmotic solution and dehydration was conducted.

Each OD process was carried out in an IS-14S ultrasonic bath produced by INTERSONIC (Olsztyn, Poland) in a stabilized temperature of 308 K for 120 min. Two different variants of the process were analyzed: OD and USOD. In the case of USOD, US at a low frequency of 25 kHz was used. Since US was generated into a water bath and then transferred to the solution in beakers, this method should be considered as indirect.

The kinetics of OD were analyzed on the basis of water loss (WL), solid gain (SG), and moisture content (MC). WL is the amount of water transferred from the fruit into the solution due to osmotic pressure difference, expressed in kg/kg (wet basis). SG is the amount of solute solid, in kg/kg (wet basis), infused into the material from the solution as a countercurrent flow. MC expresses the ratio of the moisture mass to the initial mass of the sample. The following equations were used to calculate SG, WL, and MC:

(1) $$\mathrm{WL}=\left[\left(m_{i}-m_{p}\right)+\left(s_{p}-s_{i}\right)\right]/m_{i}$$

(2) $$\mathrm{SG}=\left(s_{p}-s_{i}\right)/m_{i}$$

(3) $$\mathrm{MC}=\left(m-s_{i}\right)/m_{i}$$

where *m*_i_ is the initial mass of the sample, *m*_p_ is the mass of the processed (osmotically dehydrated) sample, *s*_i_ is the mass of dry matter in the fresh material, *s*_p_ is the mass of dry matter in the processed (osmotically dehydrated) samples, and *m* is the mass of the sample at a given time of the process.

Dry matter was determined through drying in a convective dryer at 105 °C until weight became constant. The weight loss of kiwifruit samples during OD was measured every 15 min of the process. In order to do this, the slices were pulled from the osmotic solution, drained on absorbent paper and weighed on a Vibra AJH-2200CE laboratory balance (precision 0.01 g) produced by Shinko Denshi (Tokyo, Japan).

### 2.3 Drying operation

**Fig. 1:**
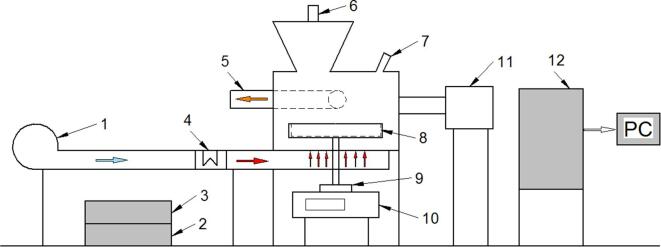
Schematic drawing of the hybrid dryer: 1-blower, 2-Airborne Ultrasound System (AUS) controller, 3-AUS amplifier, 4-heater, 5-air outlet, 6-AUS transducer, 7-pyrometer, 8-rotatable pan, 9-pan drive, 10-balance, 11- microwave feeders, 12-control unit.

All drying processes were carried out in a laboratory-scale hybrid dryer (Fig. 1) designed and constructed by PROMIS-TECH (Wrocław, Poland).

The hybrid dryer presented in Fig. 1 uses three different drying techniques: hot air convection (CD), microwaves (MW), and US. The air for CD is drawn from the surroundings and pumped to the electric heater (Fig. 1.4, max. power 2 kW) by the blower (Fig. 1.1), and after being heated, passes directly to the drying chamber. The parameters of the drying agent, such as temperature and air velocity, are controlled automatically and can be set within the range from 30 °C to 90 °C and from 0.1 to 2 m/s, respectively. The actual values of temperature, air velocity, and relative humidity are measured constantly during drying with the use of hot-wire anemometer HD29371TC1.5 (precision 0.1 °C, 0.01 m/s) and humidity sensor HD4817ETC1.5 (precision 0.01%) produced by Delta OHM (Italy).

Low frequency (26 kHz), high-power, US is generated by the Airborne Ultrasound System (AUS) (Fig. 1.2, 1.3, 1.6) produced by Pusonics S.L. (Spain). The AUS works with a focalized acoustic field, which means that the intensity of the waves converges with increasing distance from the transducer, and at about 420 mm from the radiator (it attains its maximum at 160–170 dB). The distance between the emitter and the sample was fixed at 415 mm to ensure that the sample stayed within the focus area during the drying process. The intensity of the US can be controlled by adjusting the effective power delivered to the generator in a range from 1 to 200 W.

Dehydrated samples made using OD or USOD were placed on a special perforated rotatable scale pan (Fig. 1.8) and dried. The weight loss of the material being dried was measured automatically throughout the whole operation using a PS 6000.R2 laboratory balance (Fig. 1.10, precision 0.01 g) produced by Radwag (Poland). The temperature of the sample’s surface was measured constantly with a CT LT15 pyrometer (Fig. 1.7, precision 0.1 °C) produced by Optris (Germany). The whole drying procedure proceeded automatically and was controlled by an industrial PLC driver (Fig. 1.12) produced by WAGO (Poland). All the drying parameters were recorded at a predetermined time interval (every 5 min) and stored in the data acquisition software accompanying the drying system.

Two variants of drying were tested – CD and US-assisted CD (CDUS). In both cases, the air temperature was 333 K and the air velocity was 2 m/s. The US power used during the CDUS processes was 200 W. Each drying experiment was carried out until the kiwifruit samples attained the final moisture content *MC_f_* of 0.1 kg/kg.

On the basis of the mass measurements, the kinetics of drying were determined and presented as a plot of the relative moisture content (moisture ratio, MR) against the drying time. The relative moisture content was calculated in accordance with the following equation:

(4) $$\mathrm{MR}=\left(\mathrm{MC}-MC_{f}\right)/\left(MC_{i}-MC_{f}\right)$$

where *MC* is the current moisture content (at a given time in the process), *MC_f_* is the final moisture content (assumed constant and equal to 0.1 kg/kg wet basis) and *MC_i_* is the initial moisture content. The initial moisture content was determined using a moisture analyzer (XM120; Precisa, Switzerland, precision 0.01%) and on average equaled 83.78 ± 2.24% (wet basis).

### 2.4 Product quality assessment

The quality of raw material and dried products was analyzed through measurement of color change, water activity (*a*_w_), carotenoid content, and polyphenol content.

The color of the samples was measured with a Konica Minolta CR400 colorimeter and expressed in the *CIE*Lab color space. For this purpose, three randomly chosen samples were ground in an A11 Basic laboratory mill produced by IKA (Germany) to obtain a homogeneous powder and placed in special measuring sample holders. Next, 30 measurements of tristimulus color coordinates (L*, a*, and b*) were carried out and averaged. L* indicates lightness, and a* and b* are the chromaticity coordinates that indicate color directions: from red to green (a*) and from yellow to blue (b*). The average total color change (dE) was calculated using the following equation:

(5) $$\mathrm{dE}=\sqrt{{\left(L_{i}^{*}-L_{d}^{*}\right)}^{2}+{\left(a_{i}^{*}-a_{d}^{*}\right)}^{2}+{\left(b_{i}^{*}-b_{d}^{*}\right)}^{2}}$$

where *i* denotes the fresh sample and *d* denotes the processed sample.

Water activity (*a*_w_) was measured for fresh and dry samples using the LabMaster-aw Standard *a*_w_ meter (precision 0.001) produced by Novasina AG (Lachen, Switzerland). Fresh and dry samples were placed in special measuring vessels in a thermostatic chamber of the *a*_w_ meter. The temperature inside the chamber was fixed at 298 K. The measurement of *a*_w_ for the dried product was conducted after at least a 24-hour incubation period in a desiccator since the moisture profile in the dried samples needed to be aligned after processing.

Acetone extraction of carotenoids was carried out according to the method described by Buret [20]. The carotenoid content was determined using RP-HPLC-DAD (Agilent Technologies 1200 Rapid Resolution, Waldbronn, Germany) equipped with a Poroshell 120, SB-C18 column (Agilent Technology Inc, Plo Alto, USA). The mobile phase was acetonitrile containing 0.5 g/L of triethylamine (solvent A) and methanol: ethyl acetate (55:45 v/v; solvent B) (Sigma Aldrich Chemie GmbH, Steinheim, Germany) in a gradient from 95:5 to 60:20 in 20 min, the latter proportion being maintained over 40 min. The flow rate was 0.5 mL/min. The chromatogram was recorded at 454 nm [21]. Carotenoids were quantified based on β-carotene.

Polyphenol extraction was conducted according to the procedure described by Vallejo et al. [22]. Polyphenol content was determined with the same equipment as was used to determine carotenoid content. The mobile phase was 60 g/L acetic acid in 2 mM sodium acetate (solvent A; Chempur, Piekary Śląskie, Poland) and acetonitrile (solvent B; Sigma Aldrich Chemie GmbH, Steinheim, Germany). The system was run with a gradient program: 0–15% B in 15 min, 15–30% B in 25 min, 30–50% B in 5 min, and 50–100% B in 5 min. The flow rate was 1 mL/min. The phenolics were quantified at 280 nm, 320 nm, and 360 nm using the external standard method. Gallic acid, chlorogenic acid, catechin, and quercetin were used as a standard.

### 2.5 Statistical analysis

Statistical calculations were performed using Statistica ver. 12 software produced by StatSoft (StatSoft, Tulsa, Oklahoma, USA). All analyses were carried out in triplicate. Analysis of one-way variance (ANOVA) and Tukeýs multicomparison test was performed. Statistically significant differences were reported at p ≤ 0.05.

## 3 Results and discussion

### 3.1 Kinetics of osmotic dehydration

**Fig. 2:**
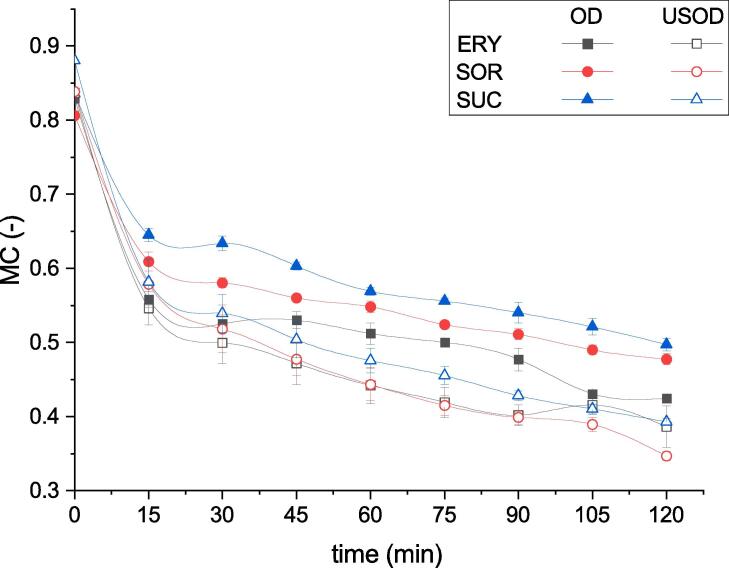
Plot of moisture content (MC) (wet basis) of samples during osmotic dehydration (OD) or ultrasound-assisted osmotic dehydration (USOD) in different solutions: erythritol (ERY), sorbitol (SOR), and sucrose (SUC).

Fig. 2 presents the change in MC of the kiwifruit slices during osmotic dehydration in different hypertonic solutions of ERY, SOR, and SUC, carried out with USOD and without ultrasound (OD). As can be seen, the first 15 min of the process were the most effective as these had the highest water loss. The reduction in MC depended on the type of active substance applied during OD as well as USOD.

During OD, the lowest final MC was observed for samples that had been dehydrated in ERY (42.47 ± 0.33%), whereas for other sugars, this parameter was slightly higher (47.76 ± 0.74% for SOR and 49.72 ± 0.82% for SUC). For USOD, the lowest final MC was recorded for samples that had been dehydrated in SOR (34.67 ± 0.60%), whereas for ERY and SUC, the final MC was slightly higher and amounted to 38.65 ± 2.77% and 39.31 ± 0.44%, respectively. Comparing the impact of US on the dehydration process, it can be seen that the samples processed with USOD had a significantly lower final MC (after 120 min of pretreatment) than samples processed without the use of US. The largest differences between the final MC of samples processed with OD and USOD (at 120th min, Fig. 2) were observed for SOR and SUC (27% and 21%, respectively) and a smaller difference was noted for ERY (9%).

Similarly, Nowacka et al. [23] observed that USOD is an effective method of moisture reduction in kiwifruit slices. The authors of this study presented SEM photos that suggested that greater mass exchange could be caused by the creation of microchannels as well as visible changes in tissue structure. It is worth emphasizing that at the end of osmotic pretreatment (around 120 min), the kiwifruit slices started to break up as the flesh was beginning to loosen.

The values of the kinetic parameters of WL and SG are presented in Table 1. The change (%) denotes the increase or decrease of the analyzed parameter observed for USOD compared to the reference process (OD).

**Table 1:** Values of water loss (WL) (a) and solid gain (SG) (b) of samples after 120 min of osmotic dehydration (OD) or ultrasound-assisted osmotic dehydration (USOD) in a solution of erythritol (ERY), sorbitol (SOR), or sucrose (SUC).

	WL∙10^3^ (kg/kg wet basis)			SG∙10^3^ (kg/kg wet basis)		
Osmotic agent	OD*	USOD*	change (%)	OD*	USOD*	change (%)
ERY	404.3 ± 3.3	466.1 ± 7.3	15%	167.8 ± 0.1	185.2 ± 0.4	10%
SOR	325.8 ± 4.7	488.7 ± 1.7	50%	114.4 ± 5.0	145.4 ± 0.9	27%
SUC	338.5 ± 8.2	487.3 ± 4.5	44%	115.7 ± 4.5	152.6 ± 0.6	32%

* mean ± standard deviation.

Analyzing the results obtained for particular osmotic agents, it can be stated that for samples dehydrated in SOR and SUC, the values of WL and SG are very similar for each type of pretreatment (OD or USOD). That means that both sugars behave in the same manner during long-term dehydration. Generally, the samples dehydrated in ERY had higher values of WL and SG compared to samples dehydrated in SOR and SUC. This can be caused by the differences in the structural and physicochemical properties of the sugars.

Irrespective of the sugar, higher WL and SG were found for USOD than OD. This may be due to the changes inside the fruit structure caused by cavitation and microstreams (effects of US) and osmosis. These changes are similar to those observed during fruit ripening, i.e. cell wall swelling and dissolution of the middle lamella [24], making the material more susceptible to mass transport in both directions. As can be seen in Table 1, the biggest change in WL was observed for samples dehydrated in SOR (50%) and the smallest for samples in ERY (15%). For SG, the biggest change was for samples dehydrated in SUC (32%) and the smallest for those in ERY (10%).

On the basis of the results obtained in the first part of the research, the effective time length for OD was determined. The first 30 min of the process were the most favorable because during this period, water loss from the kiwifruit was the greatest and the dehydrated fruit remained intact (did not disintegrate). The results of WL and SG after 30 min of pretreatment are presented in Table2. The change (%) denotes the increase or decrease of the analyzed parameter observed for USOD compared to the reference process (OD).

**Table 2:** Water loss (WL) (a) and solid gain (SG) (b) of samples after 30 min of osmotic dehydration (OD) or ultrasound-assisted osmotic dehydration (USOD) in a solution of erythritol (ERY), sorbitol (SOR), or sucrose (SUC).

	WL∙10^3^ (kg/kg wet basis)			SG∙10^3^ (kg/kg wet basis)		
Osmotic agent	OD*	USOD*	change (%)	OD*	USOD*	change (%)
ERY	243.3 ± 2.0	245.7 ± 3.8	1%	75.9 ± 0.5	110.9 ± 0.2	46%
SOR	214.6 ± 3.1	240.0 ± 0.8	12%	51.6 ± 2.3	88.8 ± 1.2	72%
SUC	141.3 ± 3.4	200.9 ± 1.9	42%	28.1 ± 1.1	53.4 ± 0.2	90%

* mean ± standard deviation.

The results in Table2 show that the better substances for dehydrating the kiwifruit are sugar alcohols, i.e. SOR and ERY. In both variants of the process (OD and USOD), the samples processed with sugar alcohols had a visibly higher WL compared to those processed with SUC. The increase in WL was accompanied by a simultaneous increase in SG, which is not always beneficial. In the case of SOR, excessive consumption may cause a laxative effect. However, for this polyol, a smaller infusion of the soluble solid (lower SG) compared to ERY was observed. In turn, ERY is considered to be a low-digestible compound that does not cause gastric discomfort. Hence, both ERY and SOR are good alternatives to common sugar.

Comparison of processes carried out with and without US assistance also brought interesting observations. For samples dehydrated in SUC, the application of US had a distinctly positive effect on WL. US also had a positive effect on the WL of samples dehydrated in SOR, but this was visibly smaller than for those in SUC. For ERY, the difference in WL between OD and USOD was almost imperceptible (see Table2).

It was also found that US significantly influenced the infusion of soluble solids. Differences between the SG observed for OD and USOD processes were meaningful. The trend of changes was the same for both types of processes: the lowest SG value was recorded for samples processed in SUC, the intermediate in SOR, and the highest in ERY. This may be due to the structure of the particular compounds and their physio-chemical properties.

The SG value may be affected by both the solute particle size and the osmotic pressure difference. The smaller molecules easily diffuse into the sample surface and penetrate between the wall and the cell membrane. On the other hand, a greater difference in osmotic pressure causes a larger flux of water (from the cell to the solution), which is usually accompanied by increases in the counter-flux of solute solid (from the solution to the cell). Taking into account the molar mass and density of the substances used in the study, it may be stated that they decrease in the following way: SUC > SOR > ERY. Because the solutions were prepared by weight, and the same mass was used for each agent, the osmotic pressure depended on the molar mass of the substance being dissolved. The lower the molar mass, the greater the amount of moles in the solution (higher molar concentration), and thus the higher the osmotic pressure [25]. In this way, the osmotic pressure of the solution was as follows: SUC < SOR < ERY, which stays in good agreement with the obtained results.

### 3.2 Kinetics of drying

**Fig. 3:**
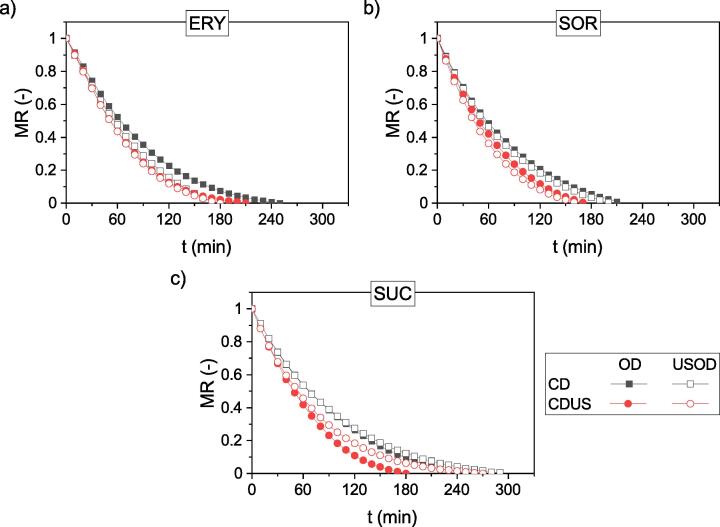
Plots of the moisture ratio (MR) for samples subjected to convective drying (CD) or ultrasound-assisted convective drying (CDUS) after osmotic dehydration (OD) or ultrasound-assisted osmotic dehydration (USOD) in a solution of erythritol (ERY) (a), sorbitol (SOR) (b), or sucrose (SUC) (c).

In the second part of the process, the samples initially dehydrated using OD or USOD were subjected to drying in the laboratory hybrid dryer. Fig. 3 presents the plots of MR against time (t).

Table3 shows the changes (%) in drying time for each type of process. This table is divided into two parts. In the first part, the change (%) denotes the increase or decrease in the drying time of samples pretreated with USOD compared to those processed with OD for a particular method of drying (CD or CDUS). Thus, the influence of US during OD on the drying time can be analyzed. In the second part of Table3, the change (%) denotes the increase or decrease of the time of CDUS compared to the time of CD for samples pretreated with OD or USOD. Thus, the influence of US during drying can be analyzed.

**Table 3:** Comparison of changes in drying time (%) for each type of process.

	change (%) USOD relative to OD		change (%) CDUS relative to CD	
osmotic agent	CD	CDUS	OD	USOD
ERY	−32%	−14%	−16%	6%
SOR	−5%	−6%	−19%	−20%
SUC	16%	50%	−28%	−7%

Comparing the curves obtained for individual drying variants (CD versus CDUS), it can be seen that regardless of the pretreatment process (OD or USOD) and the type of osmotic agent, the drying time, i.e. achieving the final moisture content, is different. The use of US during drying usually shortened the drying time. The quantitative effect, however, depends on both the type of osmotic agent and the type of pretreatment.

Analyzing the impact of US applied during osmotic pretreatment on the drying kinetics, it can be stated that for samples dehydrated in ERY and SOR, the impact of US is positive (shorter drying time for samples processed with USOD compared to OD), whereas for samples dehydrated in SUC, the impact of US is negative (longer drying time of samples processed with USOD compared to OD). For samples in ERY, the differences were more distinct and equalled 32% during CD and 14% during CDUS, whereas for samples dehydrated in SOR, these differences were smaller and close to each other, i.e. 5% for CD and 6% for CDUS (Table3). For samples dehydrated in SUC with US assistance (USOD), an extended drying time was observed compared to those dehydrated without US – 16% longer for CD and 50% for CDUS (Table3).

The obtained results may arise from the changes in the increase of SG that occurred during OD. The highest values of SG were observed for samples processed in ERY (Table2); however, the difference between the values for the OD and USOD processes was in this case the lowest and amounted to 46%. For the two other osmotic agents, the SG values were lower (Table2), but the difference between the values obtained for OD and USOD was significantly higher and amounted to 72% for samples dehydrated in SOR and 90% for those processed in SUC.

During the drying of osmotically dehydrated samples, the largest difference in drying time (CD versus CDUS) was observed for those processed in SUC (Table3), i.e. for those for which the lowest SG during OD was recorded. In addition, the lowest difference in drying time was observed for samples processed in ERY, where the highest SG was stated (Table2). This phenomenon is particularly visible in samples dehydrated with ultrasonic support (USOD), for which a significantly higher SG value was observed. The drying time in the CDUS process for samples dehydrated in SUC was only 7% shorter than the drying time for CD (Table3). On the other hand, for samples processed in ERY, for which the highest SG value was recorded (see Table2), the drying time of CDUS was 6% longer than that of CD. A different result was obtained for samples dehydrated in SOR. In this case, the CDUS drying time was 20% shorter than the drying time of CD (Table3), which is an even better result than those for samples pretreated without ultrasonic support (OD). Such a result is surprising because, as for the other osmotic agents, the SG value for samples pretreated in SOR was definitely higher for USOD. The reason for this discrepancy is not clear and needs to be analyzed.

To explain the decrease in ultrasonic drying efficiency for samples with a higher SG value, the surface effect of the mechanical waves should be considered. Because US is effective for liquid atomization, it has been utilized in many applications where small droplets need to be produced. During drying with US, atomization of the moisture/water on the surface also occurs, which leads to its rapid drying. If the surface is coated with a solution of sugar or salt, then after solvent evaporation/atomization, these soluble solids settle on the surface and create something like a crust. This crust may greatly hamper the transport of moisture, thus slow down the whole drying process. The authors of this paper have already stated in previous studies that an excessive SG increase during OD may negatively affect the drying rate [5], [26], [27].

Taking into account only the type of osmotic agent, it can be found that the shortest drying time occurred for samples dehydrated in SOR, where the average drying time equaled 185 min. Samples processed in SUC took significantly longer to dry, and the average drying time exceeded 240 min.

The positive effect of US on drying kinetics is clearly visible. As reported by Musielak et al. [18], the use of US can intensify the heat transfer and moisture/vapor migration, which in turn enhances the drying rate and reduces the total drying time or notably accelerates the process of OD due to cavitational pulsation [28].

**Fig. 4:**
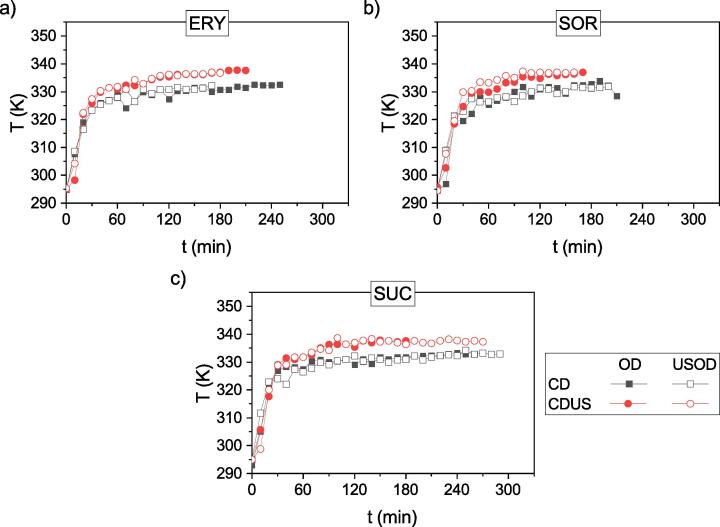
Plots of surface temperature of samples (T) subjected to convective drying (CD) or ultrasound-assisted convective drying (CDUS) after osmotic dehydration (OD) or ultrasound-assisted osmotic dehydration (USOD) in a solution of erythritol (ERY) (a), sorbitol (SOR) (b), or sucrose (SUC) (c).

The effect of US is also visible on the temperature curves of the kiwifruit samples (Fig. 4).

As shown in the temperature plots, CD and CDUS heated the surface of kiwifruit in different ways. Samples dried using CDUS heated up faster and attained higher temperatures compared to those dried using CD. Moreover, after about 60 min, the material was heated above the air temperature, and as the drying progressed, the difference in temperature became larger. The highest difference in temperature between samples dried with CDUS and CD was about 6.5 K. This is known in the literature as a “heating effect” that results from the material absorbing the acoustic energy [29]. Then, due to the heating effect, the temperature of the material increases to higher than that of the drying agent temperature. A similar temperature rise has been observed for raspberries [30] and strawberries [31] during CDUS (200 W).

As the temperature curves of the samples pretreated with OD and USOD dried by CD and those dried by CDUS are characterized by the same trend, it is appropriate to exclude here the influence of the difference in SG (see Table2).

The faster heating of the surface of the material being dried with the use of US may also be an effect of the atomization of surface moisture. The presence of water on the surface of the material being dried causes its temperature to increase slower or even to remain constant (equal to the wet-bulb temperature under the given conditions). This is usually observed in the first drying period and is due to the high heat capacity of water and the latent heat of evaporation. The energy supplied by the drying agent (hot air) is utilized to heat the water and evaporate it. When the moisture is removed from the surface, the delivered energy (heat) heats the surface, causing the temperature to rise [32]. Removal of moisture from the surface takes place in the second drying period, after exceeding what is known as the critical moisture content, however, it can also be triggered in the first drying period, e.g. by using ultrasound that may disperse surface moisture through atomization. As a result, drying is more effective and the surface of the material heats up faster.

### 3.3 Product quality

**Fig. 5:**
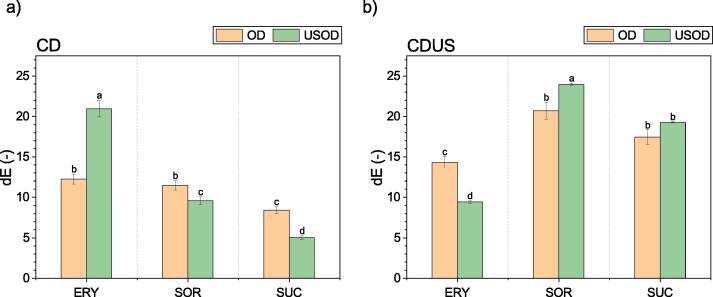
Overall color change (dE) of samples after 30 min of osmotic dehydration (OD) or ultrasound-assisted osmotic dehydration (USOD) in a solution of erythritol (ERY), sorbitol (SOR), or sucrose (SUC) for convective drying (CD) (a) or ultrasound-assisted convective drying (CDUS) (b).

In order to evaluate the effect of the different drying processes on the quality of kiwifruit, the samples were subjected to quality assessment based on dE, *a*_w_, and the content of the bioactive substances of carotenoids and polyphenols. The dE values for all the samples are presented in Fig. 5.

As can be seen in Fig. 5, dE was usually higher for samples dried using CDUS than for samples dried using CD. The highest value of dE was obtained for samples subjected to USOD in SOR solutions and CDUS: 23.96 ± 0.12. In turn, the lowest dE was observed for kiwifruit subjected to USOD in SUC solutions and CD: 5.01 ± 0.25. There is also a visible trend for the majority of the samples: those subjected to USOD had less discoloration after CD and more discoloration after CDUS. However, an opposite relationship was observed for samples dehydrated in ERY. Samples dehydrated in ERY and subjected to USOD and CD showed a higher dE than those subjected to OD. Such a color change (dE = 20.96 ± 1.05) may be due to a higher SG (see Table2) and the strong crystallization of ERY on the sample surface during osmosis and drying. As seen in Fig. 5, the use of OD on samples dehydrated in ERY resulted in similar dE values for both CD and CDUS, while the use of USOD meaningfully changed this parameter.

Such changes in color may result from the use of osmotic pretreatment. The semipermeable membrane in plant cells may not assure complete isolation from the surroundings, and during the osmotic dehydration process, minerals, vitamins, and natural dyes may diffuse into the solution and cause a partial loss of color. The use of US intensifies this phenomenon while simultaneously enhances the effectiveness of pretreatment and partially damages the cell membrane. Chlorophyll is responsible for the green color in kiwifruit. It is a temperature-sensitive compound that decreases in amount as the fruit ripens. Thus, changes in tissue structure caused by OD as well as the US treatment and hot air drying affect the dE. In our study, chlorophyll may have transformed to pheophytin (olive-brown), as described by Weemaes et al. [33].

**Fig. 6:**
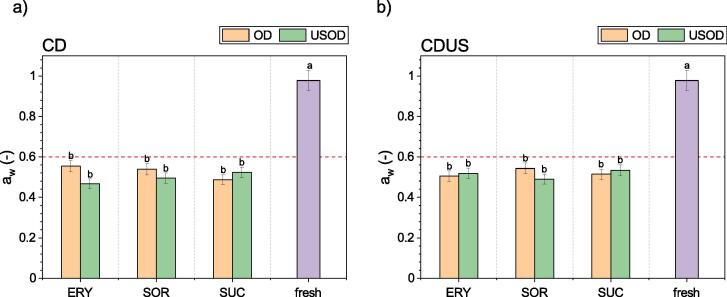
Water activity (aw) of samples after 30 min of osmotic dehydration (OD) or ultrasound-assisted osmotic dehydration (USOD) in a solution of erythritol (ERY), sorbitol (SOR), or sucrose (SUC) for convective drying (CD) (a) or ultrasound-assisted convective drying (CDUS) (b).

The next important quality indicator evaluated in these studies was *a_w_*. Water availability is often measured and expressed in terms of *a*_w_, which takes a value from 0 (a completely dry sample) to 1 (pure water). The higher the value of *a_w_* in a food product, the lower its shelf-life – spoilage processes occur faster and microorganisms such as bacteria, fungi, yeast, and mold grow more easily. The values of *a*_w_ for fresh and dry kiwifruit are shown in Fig. 6.

The fresh kiwifruit sample had a high value of *a_w_*: 0.978 ± 0.574, which is specific to fruit and vegetables. The value of *a_w_* for the dried samples (Fig. 6) revealed no significant difference between kiwifruit dehydrated with USOD or OD, or those dried using CD or CDUS. The average *a*_w_ for all dry samples was 0.514 ± 0.205.

Similarly, cherry fruit pretreated with USOD in a 60% aqueous solution of glucose and then dried using CD obtained an average *a_w_* value of about 0.5 [7]. This kind of value indicates that the products obtained are microbiologically stable, i.e., are protected against the microbial growth of mold, yeast, and bacteria. Water activity directly affects the growth of microorganisms, and most of them cannot grow at an *a_w_* value below 0.6. Therefore, *a*_w_ is a critical quality and shelf-life parameter of food consumption [34], [35].

Moreover, increased sugar content, due to osmotic pretreatment, may also have a beneficial influence on the microbial stability of the products. As reported by Gianotti et al. [36] in their studies on osmotically treated kiwifruit slices, the use of high-concentration sugar solutions (40–65%) creates a barrier limiting the growth and adhesion of microorganisms on the sample surface, even in a material with an *a*_w_ value of 0.85. This may result from the reduced mobility of microorganisms due to higher surface viscosity.

The next very important quality factor analyzed in these studies was the content of the bioactive compounds of carotenoids and polyphenols. In order to assess the effectiveness of USOD and CDUS compared to OD and CD, the retention of valuable phytonutrients contained in the kiwifruit samples was evaluated. The results of chemical analysis of different carotenoids and polyphenols are presented in Tables 4 and 5, respectively.

**Table 4:** Retention of carotenoids (%) for dried samples.

Drying method	Osmotic pretreatment	β-Cryptoxanthin* (%)	Antheraxanthin* (%)	Lutein* (%)	Total* (%)
*raw material (µg/100 g d.m.)*		*62 ± 6*	*10 ± 2*	*303 ± 13*	*375 ± 20*
CD	ERY OD	nd	nd	33 bc	27 b
	ERY USOD	nd	nd	22 a	18 a
	SOR OD	nd	90 c	64 ef	54 ef
	SOR USOD	34 a	nd	56 e	51 e
	SUC OD	32 a	70 b	44 d	41 de
	SUC USOD	26 a	nd	43 d	39 cd
*raw material (µg/100 g d.m.)*		*nd*	*10.2 ± 0.4*	*328 ± 10*	*338 ± 10*
CDUS	ERY OD	nd	97 cd	60 ef	62 fg
	ERY USOD	nd	99 d	58 ef	59 fg
	SOR OD	nd	62 b	67 f	67 g
	SOR USOD	nd	nd	41 cd	40 d
	SUC OD	nd	48 a	47 d	47 de
	SUC USOD	nd	nd	32 bc	31 bc

* retention of carotenoids (%) relative to the raw material; nd – not detected; means within columns marked by the same letter do not differ significantly.

**Table 5:** Retention of polyphenols (%) for dried samples.

Drying method	Osmotic pretreatment	Hydroxybenzoic acids* (%)	Procyanidins* (%)	Caffeic acid* (%)	p-Cumaric acid* (%)	Quercetin* (%)	Kaempferol* (%)	Total* (%)
*raw material (µg/100 g d.m.)*		*39.1 ± 0.7*	*47.2 ± 0.9*	*65 ± 2*	*10.7 ± 0.3*	*5.0 ± 0.2*	*1.9 ± 0.2*	*169 ± 2*
CD	ERY OD	56 b**	59 a	97 fgh	59 a	67 ab	97 ab	73 c
	ERY USOD	41 a	89 cd	44 ab	63 ab	70 abc	112 b	59 b
	SOR OD	72 cd	66 ab	106 h	68 ab	90 de	113 b	84 ef
	SOR USOD	40 a	67 ab	58 bc	61 ab	57 a	90 ab	57 b
	SUC OD	77 cde	80 bcd	69 cd	107 d	75 bcd	96 ab	77 cde
	SUC USOD	35 a	55 a	43 a	68 ab	66 ab	108 b	47 a
*raw material (µg/100 g d.m.)*		*20.4 ± 0.1*	*65 ± 1*	*62.3 ± 0.3*	*12.7 ± 0.3*	*8.39 ± 0.01*	*3.5 ± 0.4*	*177 ± 2*
CDUS	ERY OD	103 h	77 bc	103 gh	73 ab	99 e	89 ab	88 fg
	ERY USOD	100 gh	67 ab	69 cd	106 d	94 e	91 ab	74 cd
	SOR OD	92 fgh	92 d	108 h	73 ab	88 e	77 a	96 g
	SOR USOD	65 bc	77 bc	79 de	65 ab	86 cde	80 a	74 cd
	SUC OD	86 efg	87 cd	86 ef	82 bc	75 bcd	100 ab	83 def
	SUC USOD	84 def	66 ab	91 efg	97 cd	65 ab	91 ab	78 cde

*retention of polyphenols (%) relative to the raw material; means within columns marked by the same letter do not differ significantly.

The content of carotenoids in the tested kiwifruit samples was rather low and ranged from 338 to 375 µg/100 g d.m. In reference data, the content of carotenoids was 113–3312 µg/100 g d.m. [37], [38]. Lutein, β-cryptoxanthin, and antheraxanthin were identified in the profile and accounted for 81–97%, 16%, and 2–2.9% of the total carotenoids content, respectively. Reference data showed that the carotenoids identified in kiwifruit were neoxanthin, violaxanthin antheraxanthin, lutein, zeaxanthin, β-cryptoxanthin, and β-carotene. Lutein and β-carotene were indicated as dominant in the profile, the share of which in the total content of carotenoids was largely dependent on the genotype [37], [39]. In the conducted research, both in the raw and the dried material, the profile of the tested compounds was narrower.

The application of US in the process of OD resulted in a decrease or in a not significant impact on the content of carotenoids in the dried kiwifruit slices. Loss of carotenoids caused by the use of US was observed in the samples osmotically dehydrated in ERY and dried with CD, and the samples dehydrated in SOR or SUC and dried using CDUS (9%, 27%, and 16%, respectively). Oladejo et al. [40] also noted a higher decrease in the content of carotenoids in sweet potato subjected to USOD compared to samples dehydrated without the assistance of US. A similar effect was observed by Azoubel et al. [41] in osmotically dehydrated dried papaya. In tested samples, the reason for the reduction of carotenoids due to the use of US during OD is not clear. Loss of carotenoids may be associated with changes in tissue structure or the leakage of these compounds into the solution.

More carotenoids were retained by using US during drying, but only when SOR or ERY had been used in the process of OD or USOD. The highest retention of carotenoids was recorded in samples osmotically dehydrated in ERY or SOR and dried using CDUS – 67% and 62%, respectively. For SUC, the total content of carotenoids in the kiwifruit dried using CD or CDUS did not differ significantly. However, OD samples dried using CDUS, regardless of the osmotic agent used, resulted in a shortening of the drying time (Fig. 3). It is worth noting that when ERY and SOR were used, a higher SG was found (Table2). This could have limited the oxygen access to the dried tissue.

The results of the analysis of the second group of phytonutrients – polyphenols – are given in Table5.

The content of phenolic compounds in the tested fresh kiwifruit ranged from 169 mg/100 g d.m. to 177 mg/100 g d.m. This is in accordance with literature data [42], [43], [44], [45]. The dominant phenolic compounds were caffeic acid, procyanidins, and hydroxycinnamic acids, constituting 35–38%, 28–37%, and 12–23% of the total phenolic compound content, respectively. In addition, p-coumaric acid, quercetin, and kaempherol were identified in the tested samples. A similar phenolic compound profile was identified by Park et al. [43] in four varieties of kiwifruit grown in Korea, including the Hayward variety *(Aktinidia deliciosa*). Li et al. [45] found a dominant content of chlorogenic acid and procyanidins in eight cultivars of *Actinidia chinensis*. Numerous literature data indicate that the range and abundance of bioactive compounds in kiwifruit, including phenolics, depend on geographical location and genetic variation [46], [47], [48].

The use of USOD resulted in lower retention of total phenolics compared to typical OD, or had no significant effect on the content of phenolic compounds regardless of whether CD or CDUS had been used as the drying method. Higher losses of phenolic compounds for USOD samples in comparison to OD samples may result from larger changes in the fruit tissue caused by cavitation and microstreams. These changes can have a destructive effect on cellular structures, causing loss of phenolic compounds, especially as the increase in mass exchange during the osmosis process occurs in both directions. A decrease in the content of phenolic compounds in the osmotic dehydration process caused by US assistance was noted, among others, in dried persimmon fruit and in dehydrated sliced cranberry [49], [50]. However, for the raw materials of plums, sour cherries, and apples, an increase or no effect of US on the content of the tested compounds was found [51], [52], [53]. The research of Rahaman et al. [53], Mieszczakowska-Frąc et al. [51], and Devic et al. [54] indicate that changes in the content of phenolic compounds can be influenced by many factors, i.e. the raw material, dehydration time, temperature, and applied osmotic compound.

For CD samples, the decrease in phenolic content was mainly associated with a decrease in the content of hydroxybenzoic acid derivatives (for both OD CD and USOD CD samples), procyanidins (for OD CD samples), and caffeic acid (for USOD CD samples). For CDUS, the largest losses were recorded for p-coumaric acid, especially for OD CDUS samples, and caffeic acid and procyanidins for USOD CDUS samples. In the research of Mieszczakowska-Frąc et al. [51], significant sensitivity of apple phenolic acids and a greater decrease in phenolic acid concentration was noted when using USOD compared to OD. This relationship was not observed for procyanidins. Their retention was similar in both processes.

For CDUS samples of kiwifruit slices, the retention of phenolic compounds was higher for all samples in relation to the samples dried by CD. A higher retention was noted for CDUS kiwifruit samples subjected to OD in SOR (96%) and ERY (88%) solutions in relation to the other CDUS samples. Just like in the case of carotenoids, better retention of phenolic compounds for CDUS may be due to the shorter drying time and less access to oxygen due to better tissue impregnation when ERY and SOR were used as the osmotic agents. Similarly, Fonteles et al. [55] found a higher total phenolic content in dried cashew apple bagasse puree subjected to US compared to samples not subjected to US. Do Nascimento et al. [56] explain that the smaller losses of total phenolic content in sonicated dried passion fruit peel is caused by an increase in both the effective diffusivity and the mass transfer coefficient.

The retention of individual phenolic compounds in some dried kiwifruit samples were close to 100% or above. The high retention of kaempferol may result from diversification of the internal fruit structure and the very low content of this compound in the raw material. According to the literature, phenolic content varies amongst differing parts of a kiwifruit [57], [58]. Retention of phenolic acids above 100% may be related to the application of US, which can cause changes in the structure of the fruit and lead to better extraction. In green kiwifruit, phenolic acids occur in free form, complexes bound to proteins or polysaccharides, or in polymeric form [58], [59], [60]. The literature data suggest that technological processes may lead to the oxidation of phenolic acids as well as hydrolysis of glycosidic and ester bonds, which may result in the release of free phenolic acids and, in consequence, increase their total content [61].

## 4 Conclusion

Based on the obtained results, the most effective osmotic dehydration period of kiwifruit slices was the first 30 min of the process. The effect of ultrasound during osmotic dehydration on water loss (WL) and solid gain (SG) depended on the type of osmoactive substance, and it was the most visible in sucrose. However, the highest dehydration after osmotic dehydration (OD) or ultrasound-assisted osmotic dehydration (USOD) of kiwifruit was for sugar alcohols, with a simultaneous increase of SG. In drying, the reduction in time (regardless of the method) resulting from the use of USOD was noted only for sugar alcohols. The explanation of this phenomenon requires further research. In most cases, the application of ultrasound during drying reduced the process time against the reference process (CD) from 7 to 28%. The shortest drying time occurred for samples dehydrated in sorbitol.

Referring to the quality of the dried kiwifruit slices, the use of ultrasound-assisted convective drying (CDUS) generally led to higher color degradation compared to samples dried using CD, except for samples pretreated with erythritol. For bioactive compounds, the use of USOD generally resulted in a decrease in the content of carotenoids and phenolic compounds in the dried kiwifruit slices. In the samples processed with USOD, the average retention of carotenoids and polyphenols was lower (about 10% and 19%, respectively) compared to those dehydrated using OD. Furthermore, after CDUS of osmotically treated kiwifruit, a higher content of carotenoids as well as polyphenols was observed. The average retention of carotenoids for all samples dried with ultrasound was 51%, whereas the kiwifruit dried only with hot air retained 38% on average. In turn, the average retention of polyphenols for all kiwifruit dried using CD was 66%. However, CDUS contributed to the higher retention of total polyphenol content, i.e. 82%, for all samples. It should also be emphasized that the use of sorbitol and erythritol retained more carotenoids and polyphenols compared to the use of sucrose. The study has shown that sugar alcohols can be good substitutes for common sugar (sucrose), and they can give a better quality of dry product.

## CRediT authorship contribution statement

**Joanna Kroehnke:** Conceptualization, Methodology, Investigation, Writing - original draft. **Justyna Szadzińska:** Methodology, Writing - original draft, Writing - review & editing. **Elżbieta Radziejewska-Kubzdela:** Methodology, Investigation, Writing - original draft, Writing - review & editing. **Roża Biegańska-Marecik:** Methodology, Investigation, Writing - original draft, Writing - review & editing. **Grzegorz Musielak:** Writing - review & editing, Supervision, Project administration, Formal analysis. **Dominik Mierzwa:** Writing - original draft, Writing - review & editing, Visualization.

## Declaration of Competing Interest

The authors declare that they have no known competing financial interests or personal relationships that could have appeared to influence the work reported in this paper.
